# Comparison of quick Pitt to quick sofa and sofa scores for scoring of severity for patients with urinary tract infection

**DOI:** 10.1007/s11739-022-02927-9

**Published:** 2022-01-19

**Authors:** Manuel Madrazo, Laura Piles, Ian López-Cruz, Juan Alberola, José María Eiros, Rafael Zaragoza, Arturo Artero

**Affiliations:** 1 Doctor Peset University Hospital, Universitat de València, Avda Gaspar Aguilar n 90, 46017 Valencia, Spain; 2 Doctor Peset University Hospital, Valencia, Spain; 3 Rio Hortega University Hospital, Universidad de Valladolid, Valladolid, Spain

**Keywords:** Sepsis, Prognostic scores, Outcomes, Bacteremia, Pitt score

## Abstract

Quick Pitt (qPitt), which includes temperature, systolic blood pressure, respiratory rate, cardiac arrest, and mental status, is a new prognostic score derived from the Pitt Bacteremia score. The aim of our study is to compare qPitt with quick SOFA (qSOFA) and SOFA for scoring of severity in patients with urinary tract infection (UTI). Prospective observational study of patients diagnosed with UTI. Area under the ROC curve, sensibility, and specificity to predict 30-day mortality were calculated for qPitt, qSOFA and SOFA and compared. 382 UTI cases were analyzed. Thirty-day mortality (18.8% vs. 5.9%, *p* < 0.001) and longer hospital stay (6 [1–11] vs. 4 [1–7] days, *p* < 0.001) were associated with qPitt ≥ 2. However, qPitt had a worse performance to predict 30-day mortality compared to qSOFA and SOFA (AUROC 0.692 vs. 0.832 and 0.806, respectively, *p* = 0.010 and *p* = 0.041). The sensitivity of qPitt was lower than the sensitivity of qSOFA and SOFA (70.45 vs. 84.09 for both qSOFA and SOFA, *p* < 0.001), with a specificity lower than qSOFA and similar to SOFA (60.36 vs. 82.25 and 63.61, *p* < 0.001 and *p* = 0.742, respectively). Quick Pitt had moderate prognostic accuracy and performed worse than qSOFA and SOFA scores for predicting mortality in patients with UTI.

## Introduction

Urinary tract infection (UTI) is one of the most frequent causes of bacteremia and sepsis [1], and a common cause of Emergency Department (ED) visit [2] and of frequent hospital admissions [3].

The Pitt bacteremia score (PBS) is a well-known prognostic score in patients with bacteremia and sepsis [4, 5]. Since 2016, SOFA has been accepted as the score to diagnose sepsis [6], and quick SOFA (qSOFA), a simpler score that does not require laboratory tests, was developed for scoring in non-critical care settings [7]. Both scores have been prospectively evaluated for scoring of severity for infections from several sources.

Recently, another “quick” score, derived from the PBS, quick Pitt (qPitt) has been validated for scoring outcomes in patients with Gram-negative bacteremia (GNB) [8]. In later studies qPitt has also been evaluated for scoring for severity in *Staphylococcus aureus* bacteremia [9, 10], non-bacteremic infections from different sources [11] and complications after GNB, such as acute myocardial infarction and acute ischemic stroke [12]. Although some of these studies included a significant number of UTI cases [8, 11], qPitt has not yet been prospectively studied in patients solely with UTI. Therefore, we have conducted a prospective study with patients with UTI to compare qPitt to qSOFA and SOFA for scoring of severity.

## Materials and methods

Cohort prospective observational study of patients consecutively admitted to a university hospital, diagnosed at the ED with community-acquired UTI, from January 2017 to December 2020. Cases with a negative urine culture or a clinical syndrome compatible with any other condition after reviewing the case were excluded, as well as nosocomial UTI. Epidemiological and clinical variables were collected by the authors following a protocol. All cases were reviewed by two independent researchers before being included in the study.

SOFA, qSOFA and qPitt scores were used according to their original definitions [7, 8, 13] and measured at the ED. QPitt score assigns 1 point to each variable: temperature < 36 °C, SBP < 90 mmHg or vasopressor use, RR ≥ 25 bpm or need for mechanical ventilation, cardiac arrest and altered mental status [8]. Community-acquired healthcare-associated UTI (HCA-UTI) was defined as a community onset UTI with any of the following criteria: (i) to have been admitted to an acute care hospital ≥ 48 h within 90 days previous to current hospital admission; (ii) to have received antimicrobial therapy within 90 days previous to admission; and (iii) residing in a nursing home [14, 15].

Quantitative variables were compared using Student’s *t*-test or analysis of variance (ANOVA) when the distribution was normal, or Mann–Whitney *U*-test when it was not normal. Qualitative variables were compared with *χ*^2^ test and Fisher’s exact test, considering an *α* significance level of 0.05 for all tests. All tests were two sided. If any data was missing, a normal value was attributed for the calculation. Epidat v 3.2 was used to calculate sensibility, specificity, positive and negative predictive value of the scores to predict 30-day mortality, the area under the ROC curve (AUROC) and to compare the AUROC. The statistical package SPSS version 22 from IBM for Windows was used for other statistical analysis.

The study received approval from the Clinical Research Ethics Committee of the Doctor Peset University Hospital and followed the STROBE statement.

## Results

A total of 382 UTI cases were analyzed, with a range of age from 20 to 104 years, a median age of 78.5 [70–86], and 50.3% were women. One hundred and sixty-five (43.2%) of the patients had qPitt ≥ 2, 41.9% of the patients were septic, and 37.8% had bacteremia. In-hospital mortality was 7.9%, 30-day mortality was 11.5% and median hospital stay was 5 [3–7] days. Only three patients were transferred to the intensive care unit (ICU), the three of them with qPitt ≥ 2. The empirical antibiotics used were ceftriaxone (44.2% of cases), carbapenems (20.5%), ceftriaxone + aminoglycosides (8.6%), beta-lactam/beta-lactamase inhibitor combination (7.1%), quinolones (6.3%) and others (12.5%).

A qPitt ≥ 2 was related to age, comorbidities such as dementia, urinary catheter, community-acquired healthcare-associated UTI variables such as previous hospitalization and living in a nursing home, and severity at admission (APACHE II ≥ 12, qSOFA and SOFA ≥ 2). It was also related to lactate, but not to procalcitonin or C reactive protein, nor was it related to bacteremia. Other clinical variables may be seen in Table 1.

**Table 1:** Epidemiological and clinical characteristics of patients with community-acquired urinary tract infections according to qPitt scoring

	Total *n* 382	qPitt ≥ 2 *n* 165	qPitt < 2 *n* 217	*p*
75 years or older, *n* (%)	246 (64.4)	133 (80.6)	113 (52.1)	< 0.001
Female sex, *n* (%)	192 (50.3)	92 (55.8)	100 (46.1)	0.064
Charlson ≥ 3, *n* (%)	336 (87.9)	157 (95.2)	179 (82.5)	< 0.001
Barthel < 40, *n* (%)	122 (31.9)	82 (49.7)	40 (18.4)	< 0.001
Comorbidities, *n* (%)				
Dementia	98 (25.7)	71 (43)	27 (12.4)	< 0.001
Diabetes	137 (35.9)	65 (39.4)	72 (33.2)	0.236
COPD†	47 (12.3)	15 (9.1)	32 (14.7)	0.116
CKD‡	122 (31.9)	58 (35.2)	64 (29.5)	0.268
Cancer	80 (20.9)	33 (20)	47 (21.7)	0.706
Urinary catheter	79 (20.7)	46 (27.9)	33 (15.2)	0.003
Community-acquired health care-associated UTI╙	215 (56.3)	105 (63.6)	110 (50.7)	0.012
Previous hospitalization	124 (32.5)	67 (40.6)	57 (26.3)	0.004
Previous antimicrobial therapy	182 (47.6)	83 (50.3)	99 (45.6)	0.408
Nursing home residence	27 (12.3)	19 (11.5)	8 (3.7)	0.004
Severity scores, *n* (%)				
APACHE ≥ 12	158 (41.4)	96 (58.2)	62 (28.6)	< 0.001
qSOFA ≥ 2	97 (25.4)	69 (41.8)	28 (12.9)	< 0.001
Sepsis (SOFA ≥ 2)	160 (41.9)	101 (61.2)	59 (27.2)	< 0.001
Septic shock	37 (9.7)	27 (16.4)	10 (4.6)	< 0.001
Laboratory findings, *n* (%)				
Lactate > 2 mmol/L	144 (37.7)	77 (46.7)	67 (30.9)	0.002
CRP § > 5 mg/L	362 (94.7)	152 (92.1)	201 (92.6)	0.062
PCT ¶ > 0.5 ng/mL	79/127 (62.2)	39/62 (62.9)	40/65 (61.5)	0.981
Bacteremia (positive/total bloodcultures)	84/222 (37.8)	33/93 (35.5)	51/129 (39.5)	0.577
Polymicrobial urinary tract infection	32 (8.4)	21 (12.7)	11 (5.1)	0.009
IEAT ††, *n* (%)	85 (22.3)	42 (25.5)	43 (19.8)	0.215
Outcomes				
Hospital mortality, *n* (%)	31 (7.9)	21 (12.1)	10 (4.6)	0.011
30-days mortality, *n* (%)	44 (11.5)	31 (18.8)	13 (5.9)	< 0.001
Median hospital stay, days; mean [IQR]	5 [3–7]	6 [1–11]	4 [1–7]	< 0.001

†COPD, chronic obstructive pulmonary disease; ‡CKD, chronic kidney disease; ╙UTI, urinary tract infection; §CRP, C reactive protein; ¶PCT, procalcitonin; ††IEAT, inadequate empirical antimicrobial therapy; IQR, interquartile range

**Fig. 1:**
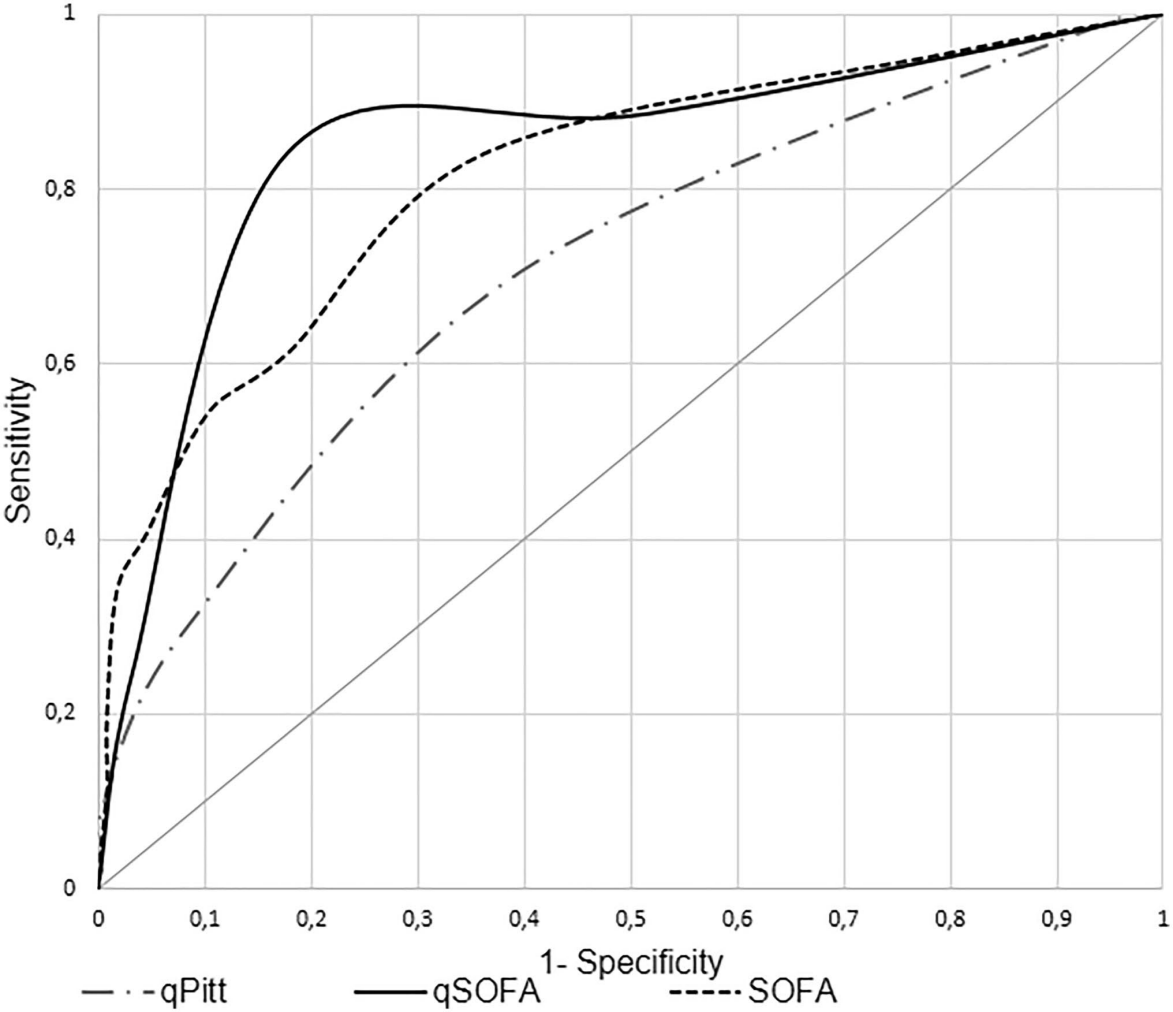
Area under the receiver operating characteristic curve of quick Pitt, quick SOFA and SOFA for 30-day mortality of patients with community-acquired urinary tract infection

Quick Pitt ≥ 2 was associated with in-hospital mortality (12.1% vs. 4.6%, *p* = 0.011), 30-day mortality (18.8% vs. 5.9%, *p* < 0.001) and longer hospital stay (6 [1–11] vs. 4 [1–7] days, *p* < 0.001). Quick Pitt had a worse performance to predict 30-day mortality compared to qSOFA and SOFA (AUROC 0.692 vs. 0.832 and 0.806, respectively, *p* = 0.010 and 0.041), as we can see in Fig. 1. The sensitivity of qPitt was lower than the sensitivity of qSOFA and SOFA (70.45 vs. 84.09 for both qSOFA and SOFA, *p* < 0.001), with a specificity lower than qSOFA and similar to SOFA (60.36 vs. 82.25 and 63.61, *p* < 0.001 and *p* = 0.742, respectively), as we can see in Table 2.

**Table 2:** Performance of qPitt, qSOFA and SOFA for 30-day mortality in patients with community-acquired urinary tract infection

	AUROC (IC 95%)	Sensitivity (IC 95%)	Specificity (IC 95%)	Positive predictive value (IC 95%)	Negative predictive value (IC 95%)
qPitt ≥ 2	0.692 (0.605–0.779)	70.45 (55.84–85.07)	60.36 (54.99–65.72)	18.79 (12.52–25.05)	94.01 (90.62–97.40)
qSOFA ≥ 2	0.832 (0.761–0.903)	84.09 (72.15–96.03)	82.25 (78.03–86.47)	38.14 (27.96–48.33)	97.54 (95.57–99.52)
SOFA ≥ 2	0.806 (0.731–0.881)	84.09 (72.15–96.03)	63.61 (58.33–68.89)	23.13 (16.28–29.97)	96.85 (94.32–99.37)
p qPitt vs. qSOFA	0.010	< 0.001	< 0.001	< 0.001	< 0.001
p qPitt vs. SOFA	0.041	< 0.001	0.742	0.085	< 0.001

Thirty-seven percent of the patients with blood cultures had bacteremia. Quick Pitt did not show differences between patients with and without bacteremic UTI (35.5% vs. 39.5%, *p* = 0.577), see Table 1. Quick SOFA and SOFA were positive in more patients with bacteremic UTI, compared to patients without bacteremia (40.5% vs. 26.8%, *p* = 0.034; 55.9% vs. 42.1%, *p* = 0.044, respectively).

qPitt, qSOFA and SOFA showed a slightly better performance for predicting 30-day mortality in bacteremic UTI than in non-bacteremic UTI (AUROC 0.702, 0.848 and 0.839 vs. 0.686, 0.810 and 0.773 for qPitt, qSOFA and SOFA, respectively). The sensitivity of qPitt in bacteremic UTI compared to non-bacteremic UTI was lower (66.67 vs. 69.23), while the specificity was higher (68.18 vs. 59.20).

## Discussion

The qPitt simplified the PBS into five binary variables and can be calculated at the bedside without the need for further laboratory tests [8]. It showed a discerning performance for predicting mortality in previous studies [9, 11], but an external validation is necessary in patients with UTI.

In our results, we found that qPitt was associated with several poor prognosis risk factors, such as age, community-acquired healthcare-associated UTI or comorbidities, as in other studies [8, 11], and severity scores, such as APACHE II and SOFA ≥ 2. QPitt was also associated with poor prognosis itself (in-hospital mortality, 30-day mortality and a longer hospital stay), as in other studies with infections from various sources [9, 11].

Different from the qPitt’s good performance described by Battle et al. both in GNB and *S. aureus* bacteremia (AUROC 0.85 and 0.82, respectively) [8, 10], our results showed a medium performance, more in line with the results of Jorgensen et al. in a study on *Enterobacteriaceae* bacteremia (AUROC 0.647, sensitivity 72.2, specificity 57.1) [16].

The performance of qSOFA in the original study of Battle et al. [8] was slightly worse than our results (AUROC 0.77, in contrast with 0.832), especially at the expense of specificity (50 vs. our 82.25). This may be the result of the better performance of qSOFA in UTI, suggested by other authors [17], who compare the performance of qSOFA in patients at the ED with respiratory infections, UTI and other infections. It is notable that both scores were not statistically compared by Battle et al. [8].

There is a considerable overlap between clinical variables included in both quick scores, as noted in Battle et al. [8]. Altered mental status is a common variable in the two scores, and the difference in systolic blood pressure and respiratory rate may be seen as subtle. However, there is enough difference to establish a significant difference in our results, with a better specificity of qSOFA compared with the other scores, not at the expense of lower sensitivity. Therefore, the performance of the score had better results for qSOFA, which were comparable to other studies [7, 18, 19].

Henderson et al. [11] compared the performance of PBS in bacteremic and non-bacteremic infections, with similar results. However, they did not compare the performance of qPitt in non-bacteremic infections. In our results, the performance of qPitt in bacteremic and non-bacteremic UTI were also comparable. It is notable that qPitt, derived from PBS, a score for prognosis in bacteremia, was not related to bacteremia, nor had significantly better performance in bacteremic UTI.

The main strength of our study is its clinical conception. There are other studies that validated qPitt [8, 9, 11, 16], but the patients were clinically not comparable, with different sites of acquisition of the infection and source of the infection [9–11]. Our study has a homogeneous sample of patients with community-acquired UTI, not centered only in gramnegative bacteria [11, 16] or *S. aureus* bacteremia [9, 10]. The main limitation of our work is that it was carried out in a single center. All in all, we think that our findings may help the clinician to treat patients better with community-acquired UTI, with or without community-acquired health care-associated criteria.

In conclusion, patients with qPitt ≥ 2 had higher mortality and longer hospital stay. However, quick Pitt had moderate prognostic accuracy and performed worse than qSOFA and SOFA scores for predicting mortality in patients with UTI.
